# YOLO-G: Improved YOLO for cross-domain object detection

**DOI:** 10.1371/journal.pone.0291241

**Published:** 2023-09-11

**Authors:** Jian Wei, Qinzhao Wang, Zixu Zhao

**Affiliations:** Army Academy of Armored Forces, Beijing, China; TU Wien: Technische Universitat Wien, AUSTRIA

## Abstract

Cross-domain object detection is a key problem in the research of intelligent detection models. Different from lots of improved algorithms based on two-stage detection models, we try another way. A simple and efficient one-stage model is introduced in this paper, comprehensively considering the inference efficiency and detection precision, and expanding the scope of undertaking cross-domain object detection problems. We name this gradient reverse layer-based model YOLO-G, which greatly improves the object detection precision in cross-domain scenarios. Specifically, we add a feature alignment branch following the backbone, where the gradient reverse layer and a classifier are attached. With only a small increase in computational, the performance is higher enhanced. Experiments such as Cityscapes→Foggy Cityscapes, SIM10k→Cityscape, PASCAL VOC→Clipart, and so on, indicate that compared with most state-of-the-art (SOTA) algorithms, the proposed model achieves much better mean Average Precision (mAP). Furthermore, ablation experiments were also performed on 4 components to confirm the reliability of the model. The project is available at https://github.com/airy975924806/yolo-G.

## Introduction

Deep convolutional models significantly improve the precision of object detection [1–11]. However, the models are severely constrained by training data. Cross-domain object detection requires the model to fulfill the training process in a fully annotation training set and then applied in the validation from a different domain. Poor results or even degradation is likely to come following due to such a domain gap. Well, in the real world, such differences, roughly lighting conditions, weather conditions, view angles, equipment differences, etc. [12], are quite common. Furthermore, it is impossible to provide infinite training data. That is to say, gathering more data is not a reliable way to improve the ability and enhance the robustness of the model.

Addressing the cross-domain object detection problem within limited labeled training data, DAF [13] pays attention to this problem for the first time, and an improved two-stage detection model based on Faster R-CNN [10] is designed, which took an unsupervised training method to upgrade its detection ability under changing weather conditions. Following up, in [14–23] et al., the addition of guidance branches, fusion generative adversarial model, and other methods are used to gain a much better ability. In [16, 18, 24, 25], semi-supervised methods are adopted, such as adjusting the training strategy, adding the knowledge distillation mechanism [26], or introducing an iterative training way, these all intend to gradually improve the model cross-domain detection ability. Well, these methods significantly increase the budget of computation and lengthen the training time.

Compared with the two-stage detection model, YOLO has more advantages in terms of speed, precision, and application. At present, YOLO-based cross-domain detection models are developing rapidly [27–33]. Focusing on the cross-domain object detection problem, this paper takes the YOLOv5-L model as the baseline. Especially, we adopt the idea of feature alignment which is widely used in two-stage models, and add unsupervised adversarial training branches to improve the adaptive ability of the model. By adopting such a simple and efficient branch, the model gains fresh new abilities. Specifically, to realize the adaptive feature alignment and reduce the domain gap between different images [12, 13], we introduce the adversarial training mechanism into the model parameter update process. Especially, to release the burden, we use a naive three-layer classifier based on the full convolution network and global average maximum pooling, with gradient reverse [34, 35] operation. Compared with the dense linear layer, the YOLO-G just slightly increases the amount of computation, however, the precision is greatly improved. To verify the proposed model in cross-domain object detection tasks, this paper carries out extensive experiments under 6 benchmark sets. The results show that YOLO-G achieves better detection precision than a series of semi-supervised and two-stage SOTA models. The main contributions of this paper are as follows:

1. For cross-domain object detection tasks, we verify the usability of the YOLO model in cross-domain object detection tasks through comprehensive experiments. Our ablation experiments show that under the source-only condition, the YOLOV5-L model can compare with many SOTA algorithms.
2. The YOLO-G model is designed based on YOLOV5-L. A concise and efficient unsupervised adversarial training branch is added to the baseline model. In this way, we expand the scope of cross-domain model design and change the previous situation limited to two-stage models.
3. We carry out qualitative and quantitative experiments and compare 10 algorithms in 6 cross-domain benchmarks. In order to fully illustrate the credibility of the model, we also carry out ablation experiments in 2 aspects and 4 factors. Experimental results show that the proposed model has indeed achieved better detection results.

## Related work

### Object detection

As an important content of computer vision research, object detection models have developed rapidly with the support of deep learning technology in recent years. Among them, the one-stage detection models are represented by YOLO [5, 7, 27] and SSD [8]. Especially, the YOLO model has gradually developed into a rich series with fast infer speed, accurate precision, and simple deployment. The two-stage detection model, represented by Mask R-CNN [9], and Fast R-CNN [10], occupies an important position in academic research because the models adopt the process of separating ROI region generation and classification discrimination, making the model have higher plasticity. Thanks to the finer ROI search strategy, the two-stage models have higher detection precision under the same conditions. At present, with the application of the transformer, the object detection model based on the transformer [11, 36] with larger parameter scales is shining brightly and shows good development prospects.

In actual deployment scenarios, the YOLO series model has better compatibility. Therefore, considering the task requirements, application scenarios, and inference speed, this paper selects the YOLOv5-L model as the baseline and improves it to adapt to cross-domain object detection tasks.

### Cross-domain

DAF [13] creatively uses the two-stage detection model to solve the cross-domain object detection problem and proposes a processing method based on global feature alignment and target feature alignment. Inspired by it, subsequent research [14–18, 24, 37] is mostly based on the two-stage detection model. Namely a few, SWDA [38] proposes to use enhanced local features and global features as auxiliary feature alignment, GPA [39] designs a detection framework based on class feature alignment, MAF [40] is different from the feature alignment method, ATF [14] and PA-ATF [41] try to use multi-branch supervised training to improve the cross-domain object detection ability. With the deepening of related research content, there have been attempts based on one-stage detection models, such as EPMDA [28] integrates FCOS [42] modules into YOLO backbone to improve its ability to extract object features in cross-domain images, SSDA-YOLO [12] adds CUT [43] and knowledge distillation mechanism to YOLO-based cross-domain object detection model [32, 44], are also based on YOLO. Inspired by them, this paper also takes the one-stage detection model as the baseline to explore cross-domain object detection tasks. In fact, through the experimental research of this paper, it is shown that with the help of the functional branch with a limited amount of computation, YOLO-G improves the ability of YOLO in the cross-domain object detection task. Furthermore, in some tasks, the result is far higher than in some two-stage detection models.

### Cross-domain detection

Cross-domain detection tasks evolve into multiple paths in the development process, such as fully supervised, semi-supervised, and unsupervised methods. Among them, full supervision refers to the construction of full annotation data of the target domain. Through collection and annotation for data augmentation, this method achieves the purpose of improving the generalization. However, this will bring huge labor. In real application scenarios, the data of all possible scenarios cannot be collected, so this method is not advisable. The semi-supervised [15, 16, 45, 46] method proposes to use partially labeled data to guide the training process. They mainly divide the training process into multiple stages. Firstly, an initial model with fully labeled source domain data is trained, and then pseudo-labels on the target domain data are created by the pre-trained model. After setting the confidence threshold, iteratively add the detected sufficiently prepared target domain data into the training set. The gradual guidance improves the generalization ability, but this method is limited by the initial model detection ability. In many cases, the initial model is difficult to provide enough effective pseudo-labels, making it difficult to continue the iterative process. Unsupervised [13, 34, 47–50] uses the powerful self-learning ability of the deep learning model itself, by setting simple boundary conditions or even providing unlabeled data belonging to the target domain, the model can independently learn to identify the key features required of the object, which is more concise and efficient in term of implementation.

Inspired by these principles, based on the YOLOV5-L model, this paper proposes a simple and accurate cross-domain object detection model by adding unsupervised feature alignment function branches.

## Method

Firstly, we briefly introduce the YOLOV5 model, which is mainly composed of 3 parts, respectively. The backbone is responsible for feature extraction, composed of C3, CSP, and SPPF, through a variety of series and parallel forms of residual structure, to extract the feature of the input images. The neck mainly completes feature processing and fusion, using FPN [51], and CSP to achieve bottom-up and top-down feature fusion. The head module, divided into 3 layers, completes the task of detecting targets on small, medium, and large scales respectively. Compared with the two-stage model Faster RCNN, which first provides ROI by RPN and then performs classification detection, the YOLO model directly detects ROI regions on the feature map, which is more prominent in processing efficiency.

**Fig 1:**
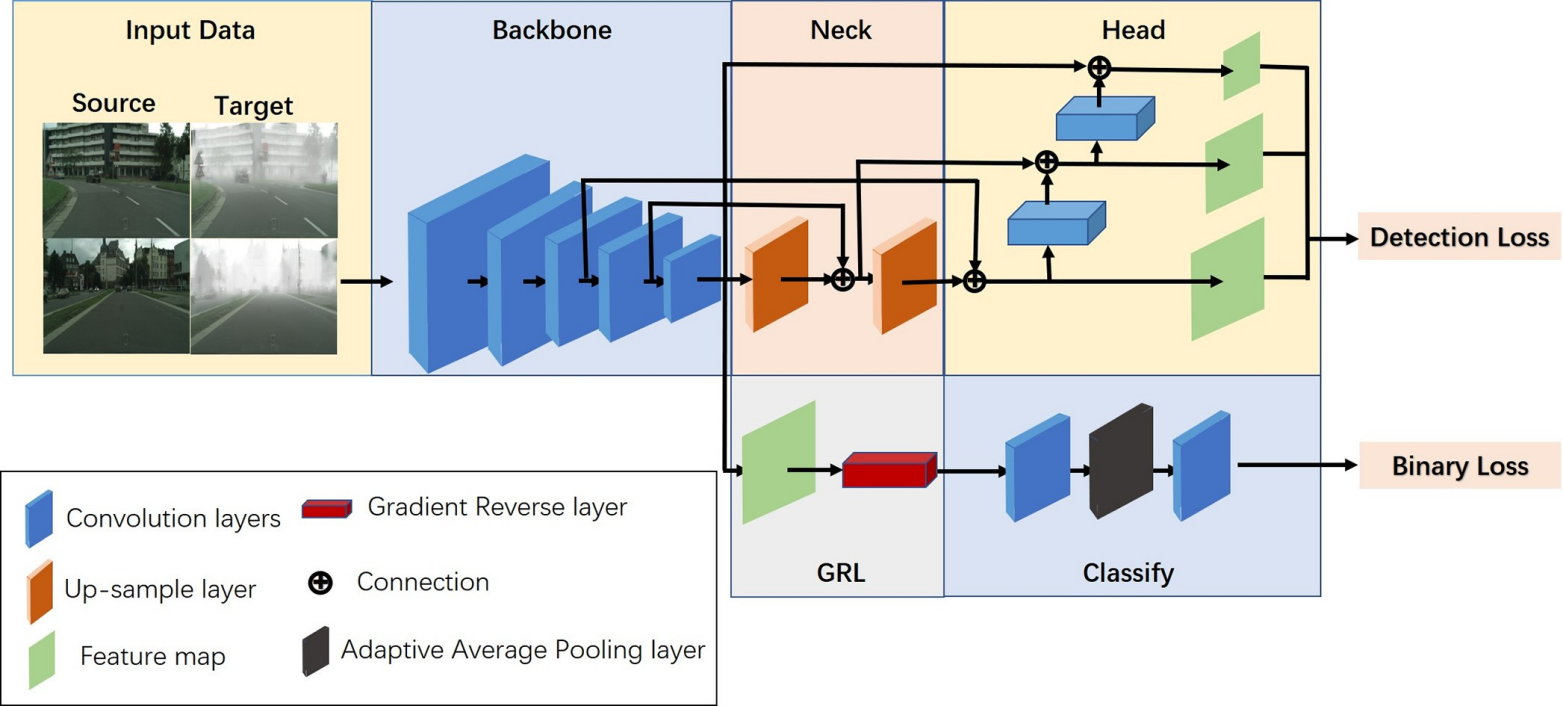
The structure of the YOLO-G. The main improvement is a simple and efficient branch is attached behind the backbone; the main modules are illustrated in the bottom-left.

As shown in Fig 1, YOLO-G adds functional branches after the backbone. Adversarial training is adapted to constrain the target images from different scenarios to achieve feature alignment in the backbone output layer. By doing so, eliminates the problem of feature inconsistency caused by scene, weather, viewing angle, or other factors, and improves the cross-domain detection precision of the object finally.

Set *S* represent the source domain, *T* is the target domain, *X*_*S*_ is one dataset collected from the source domain, *x*_*s*_(*c*,*b*)∈*X*_*S*_ is a single image, *c* is the category, *b* is the box location, which contains four scalers. *x*_*t*_∈*X*_*T*_ is a sample identified from the target domain, without category and location annotations. The cross-domain object detection task is to learn a detection model *f* from the source domain, and then directly use it for inference, which expressed as $f(x_{t})\overset{?}{\to }c_{t},b_{t}$.

The domain gap between different datasets is the inner cause of the cross-domain problem. The H-divergence [52] is used to quantitatively evaluate this difference, which is essential to measure the discriminant error of different data in the same classifier, and the formula is as follows:

$$d_{H}(S,T)=2(1-\underset{h\in H}{\min}(err_{s}(h(x_{m}))+err_{t}(h(x_{m}))))$$ (1)

where *d*_*H*_ means the calculated H-divergence, *h* is the shared classifier. In this paper, the classifier is composed of 2 convolutional layers and 1 global average pooling layer. During the training process, the label of the source domain and target domain is set to 0, 1, separately. The classifier only discriminates the domain category of the feature vector, that is *h*(*x*_*m*_)→{0,1}. *x*_*m*_ is the feature vector extracted by the backbone. *err*_*s*_, *err*_*t*_ represent the case of misclassification of the classifier. Therefore, the maximum value of misclassification is the upper bound of the difference datasets, when the classifier reaches the optimal classification ability.

In short, the main work is how to reduce the H-divergence between datasets. According to Eq (1), *x*_*m*_ is the key factor affecting H-divergence. That means the larger the H-divergence, the bigger difference for the feature, and vice versa. Therefore, this explains the model’s inability to extract consistent features, resulting in significant performance degradation in cross-domain object detection.

To reduce H-divergence, data-level alignment can be adopted, such as data augmentation technology [53, 54], through copy, paste, GAN generation, and other means to generate fake target domain images to enrich the training set. The second is pixel-level alignment [55], such as image style transfer, in cross-weather, lighting scenes. A variety of style transfer models convert the source domain to the target domain, reducing the difference in data distribution. The third is feature-level alignment [56–58], from the perspective of data distribution, feature is a low-dimensional description of the true distribution of the real data. Therefore, this paper mainly explores the method of realizing cross-domain image alignment at the feature level.

Gradient reverse [35] is an unsupervised training method first proposed in the cross-domain scene classification task, and its basic idea is to maximum-minimize the loss of the classifier. That is, to confuse the classification ability of the classifier. with the purpose to achieve the minimize *d*_*H*_. By doing so, the classifier cannot correctly classify the input features. The process is described as follows:

$$\underset{f_{b}}{\min}d_{H}(S,T)\Leftrightarrow \underset{f_{b}}{\max}\;\underset{h\in H}{\min}(err_{s}(h(x_{m}))+err_{t}(h(x_{m})))$$ (2)

where *f*_*b*_ is the feature extraction module of the object detection model, and it is the backbone of our YOLO-G.

Gradient reverse sets the opposite weights of the feature vectors during forward propagation and backward propagation. The classifier is guided to reduce the classification error when the classifier is trained forward, and the opposite weight factor is applied when the parameters are updated in the backward propagation. In an interactive way, we guide the backbone to update the heading for maximizing the classification error of the classifier.

During the training progress, the object detection model and domain classification model are updated synchronously. The object detection loss function *L*_det_ consists of three parts: object detection loss *L*_*obj*_, classify loss *L*_*class*_, and box regression loss *L*_*dc*_.

The loss function of the feature-aligned branch is the binary classify loss *L*_*dc*_ using BCE loss:

$$L_{dc}=-\sum_{batch}\sum_{i,j}\left(d_{k}\log p_{k}(i,j)+(1-d_{k})\log(1-p_{k}(i,j\right))$$ (3)

where *d*_*k*_ is the domain label of the sample with *k* index in the training batch, *p*_*k*_(*i*,*j*) is the probability of the domain classifier on the input, and a negative sign is added in front of it due to the adversarial training method.

In summary, the total loss function of YOLO-G in this paper is shown as follows:

$$L=L_{\det}+\alpha L_{dc}=\lambda_{1}L_{obj}+\lambda_{2}L_{class}+\lambda_{3}L_{box}+\alpha L_{dc}$$ (4)

where *λ*_*1*_, *λ*_*2*_, *λ*_*3*_ is set to 1.0, 0.5, and 0.05, respectively, α is the loss weights of the feature alignment branches, and through the ablation experiment, this paper sets α to 1.0.

## Experiment

### Datasets

Cityscapes [59] and Foggy-Cityscapes [60]: The two datasets contain the same number of image samples, of which 2975 training images and 500 validation images, the only difference is that the latter one is the dataset after adding fog through the rendering engine. To verify the excellent performance of the YOLO-G in cross-domain object detection, the maximum dense 0.02 is selected.

SIM 10K [61]: A dataset of vehicle driving scenes synthesized by the rendering engine of GTA5, containing 10,000 vehicle targets in various street scenes.

KITTI [62]: Autonomous driving dataset contains 7481 detailed annotated various targets.

BDD 100K [63]: Large-scale autonomous driving dataset, contains a variety of typical urban scenarios, this paper selects data under daytime conditions for experiments, including 36278 training images and 5258 validation images.

PASCAL VOC [64]: A large-scale object detection dataset, which contains 20 categories of detailed labeled images, we use VOC 2007 and VOC 2012 for experiment as in [29] with a total of 16551 images.

Clipart [45] and Watercolor: Just like VOC, both datasets contain 20 types of targets, containing 1000 and 2000 image samples, respectively.

As shown in Table 1, we summarize some characters of all the benchmark. It is clear that all the datasets have unique domain compared to each other, and this is why they are widely used in cross-domain object detection researches.

**Table 1:** Datasets features (as shown in the table, there are lots of differences between each other, especially the scene and weather play a much important role in cross-domain object detection).

Dataset	Size	Scene	Weather	Class
Cityscapes (C)	3475	City Street	Clear	8
Foggy-Cityscapes (F)	3475	City Street	Foggy	8
SIM 10K (S)	10000	Synthetic Street	Sun, Rain, Foggy	1
KITTI (K)	7481	Rural, City Street	Clear	9
BDD 100k (B)	100000	City Street, Highway	Snow, Rain, Cloud	11
PASCAL VOC	16551	Real	-	20
Clipart	1000	Synthetic	-	20
Watercolor	2000	Synthetic	-	20

### Experimental platform

The experiments are based on Ubuntu 18.04 LTS operating system, 16GB running memory, 1 Nividia RTX 3090 GPU with 24GB memory as the hardware platform, using PyTorch deep learning framework, Pycharm development platform, 11.7 Cuda, and Cudnn 8.0 acceleration environment.

### Implementation details

Considering the size and category type of the training dataset, this paper selects YOLOV5-L as the baseline, in which the input image size is unified as 640×640, the training epoch is 200, and the warm-up period is 3 epochs, and the other parameters are consistent with SSDA-YOLO. The data augmentation strategy based on mosaic is adopted to improve the detection precision of the model for small targets. We quantified the effects of mosaic and SPPF in ablation experiments. The remaining unspecified parameter settings are consistent with the original YOLO model.

### Evaluation metrics

The mean average precision (mAP) of the experiment is used as the evaluation index, and the IOU is specified to be 0.5. For the K→C, S→C experiment, since only the car target is detected, AP is used as the evaluation metric, and the threshold value of IOU is also set to 0.5.

### Result

#### Detection from Cityscapes to Foggy Cityscapes

As usual, we set Cityscapes as the source domain and Foggy-Cityscapes as the target domain. In such setting, we can test whether different cross-domain models can effectively eliminate the influence of fog when weather conditions change. It makes sense when the model extracts consistent features that are accurate enough to identify objects under fog occlusion conditions. In this paper, some of the state-of-the-art models are compared with YOLO-G, and the experimental results are shown in Table 2.

**Table 2:** The cross-domain detection results of Cityscapes → Foggy Cityscapes.

Method		person		rider		car		truck		bus		train		motor		bike		mAP	
DAF [16]		29.2		40.4		43.4		19.7		38.3		28.5		23.7		32.7		32.0	
DM [17]		31.8		40.5		51.0		20.9		41.8		34.3		26.6		32.4		34.9	
SW-DA [18]		31.8		44.3		48.9		21.0		43.8		28.0		28.9		35.6		35.1	
SC-DA [19]		33.8		42.1		52.1		26.8		42.5		26.5		29.2		34.5		35.9	
MTOR [20]		30.6		41.4		44.0		21.9		39.6		40.6		28.3		35.6		35.1	
AFAN [21]		42.5		44.6		57.0		26.4		48.0		28.3		33.2		37.1		39.6	
GPA [22]		32.9		46.7		54.1		24.7		45.7		41.1		32.4		38.7		39.5	
ViSGA [23]		38.8		45.9		57.2		29.9		50.2		51.9		31.9		40.9		43.3	
SFA [24]		46.5		48.6		62.6		25.1		46.2		29.4		28.3		44.0		41.3	
MKT [25]		43.5		52.0		63.2		34.7		52.7		45.8		37.1		49.4		47.3	
Source only		39		41.4		51.8		27.5		33.3		13.1		31.7		35.8		34.2	
YOLO-G(ours)		46.0		47.5		65.1		38.2		52.8		55.1		44.8		32.7		47.8	
Target only		61.2		52.1		77.2		47.8		62.8		60.3		50.3		46.2		57.2	

**Fig 2:**
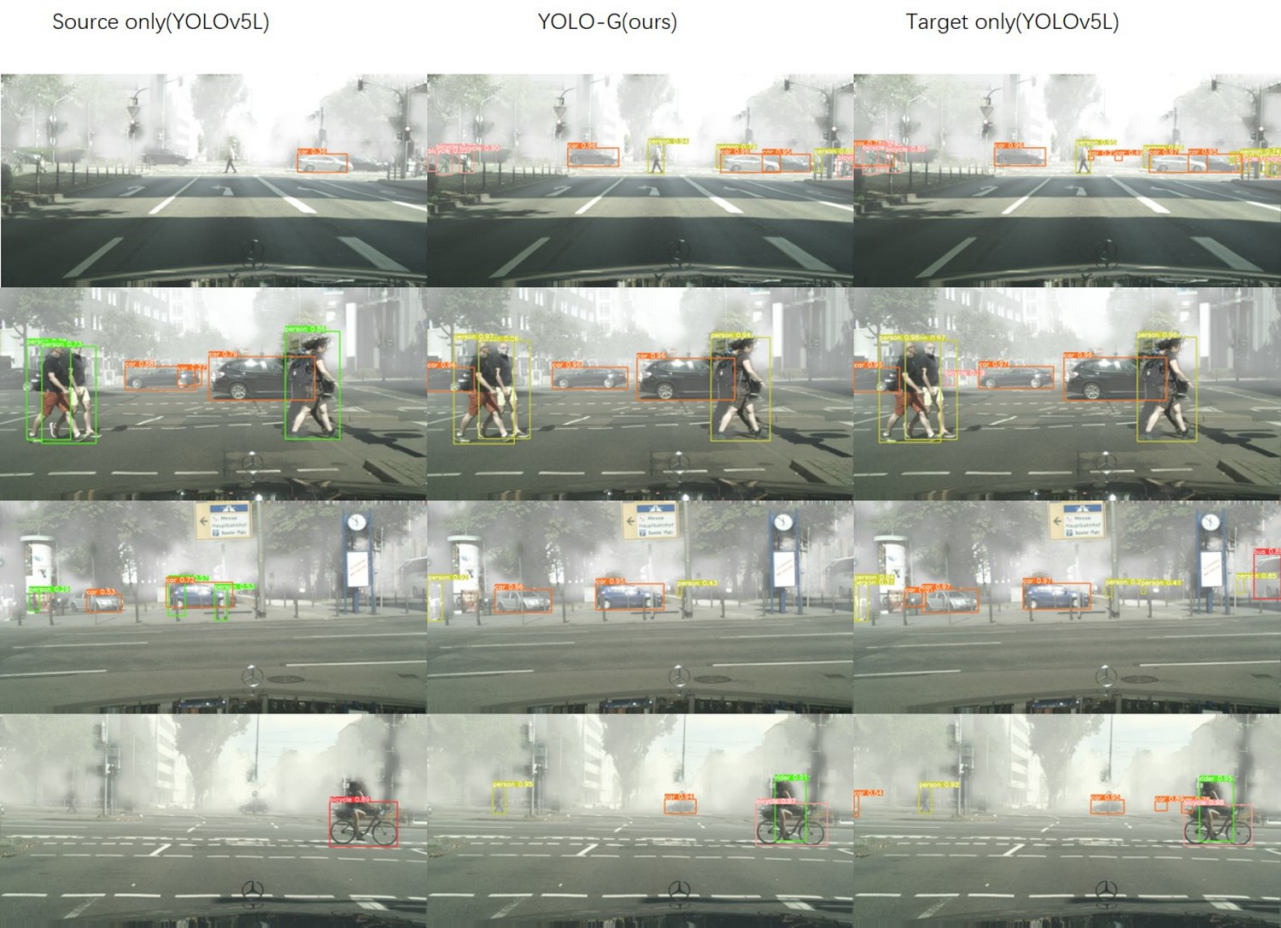
Visual detection examples using the original YOLOv5 model: left column (source only), middle column (YOLO-G), right column (target only).

It can be seen from Table 2 that compared with the method based on the two-stage detection model, YOLO-G achieves outstanding detection precision of 47.8 mAP. Furthermore, even the baseline model gets 39.9 mAP, much higher than most semi-supervised cross-domain detection models. Benefits from the rich data enhancement, SPPF used by the YOLO model, we reach the same result with SOTA such as DSS, which takes a much more complex model structure and training strategy. Regarding the role of these tricks, we conduct detailed experiments and analyses in the ablation experiment section. Although the YOLO-G model and the DSS method both reach 47.8 mAP, the difference is that YOLO-G only adopts unsupervised self-learning mode. That is to say, we do not require a substantial improvement in model structure and training strategy, nor to train in stages. In short, YOLO-G gets the highest score in 5 categories, outstanding than others, and the detection results are shown in Fig 2.

#### Detection from a different view

Different image acquisition equipment and angles will cause very different target imaging results, which is a very common target cross-domain detection problem. To test the precision of YOLO-G object detection under such conditions, this paper uses SIM 10k, Cityscapes, and KITTI datasets with different perspectives for cross-experiments, because there is only a car target in SIM 10K, AP is used as an evaluation index for this section of the experiment, and the relevant results are presented in Table 3.

**Table 3:** The cross-domain detection results on SIM 10k, Cityscapes, and KITTI.

Method	S→C	K→C
DAF [15]	41.9	41.8
SCDA [19]	45.1	43.6
SWDA [18]	44.6	43.2
GPA [22]	47.6	47.9
ViSGA [23]	49.3	47.6
SFA [24]	52.6	41.3
DM [17]	43.9	42.7
MKT [25]	50.2	44.3
AFAN [21]	45.5	--
MTOR [20]	46.6	--
SimROD [25]	52.05	--
Source only	64.0	60.5
YOLO-G(ours)	64.2	62.8
Target only	84.6	84.6

The advantages of YOLO-G in processing multi-view, multi-directional cross-domain scenes are obvious. Through the guidance of feature alignment branches, YOLO-G extracts the features of the target more accurately, ensuring that it can sample enough feature information about the target at any viewing angle, orientation, and scale to achieve effective detection. YOLO-G achieved a mAP of 64.2, and 62.8, which is at least 11.6 mAP better than the other two-stage models. However, in S→C, there is not only a difference in viewing angle but also a scene difference, YOLO-G only improves 0.2 mAP compared with the baseline model under the dual conditions of processing cross-angle and cross-virtual reality.

#### Detection from real to virtual

In many cases, there is a big difference between the virtual scene and the real environment, which can easily lead to a large deviation from the model. Combined with the results of S→C experiments, this section tests the precision of YOLO-G in processing virtual and real scene conditions. The experiment sets the VOC dataset as the source domain, Clipart, and Watercolor as the target domains, and Watercolor has 6 types of targets consistent with the VOC dataset.

It can be seen from the comparison in Table 4 that compared with the two-stage detection models, YOLO-G achieves 44.3 mAP by using autonomous feature alignment in the cross-domain detection scenario of 20 categories. It is worth noting that SSDA-YOLO is also a cross-domain detection model based on YOLOV5-L, it adopts complex training strategies such as semi-supervision, EMA, knowledge distillation, consistency loss. Under semi-supervised conditions, SSDA-YOLO gets much higher mAP, but our YOLO-G is only equipped with GRL, and achieves a slightly higher mAP compared to TIA.

**Table 4:** The cross-domain detection results from Pascal VOC to Clipart.

Method	aero	bicycle	bird	boat	bottle	bus	car	cat	chair	cow	table
SWDA [18]	26.2	48.5	32.6	33.7	38.5	54.3	37.1	18.6	34.8	58.3	17
SCL [27]	44.7	50	33.6	27.4	42.2	55.6	38.3	19.2	37.9	69	30.1
DM [17]	25.8	63.2	24.5	42.4	47.9	43.1	37.5	9.1	47	46.7	26.8
CRDA [28]	28.7	55.3	31.8	26	40.1	63.6	36.6	9.4	38.7	49.3	17.6
HTCN [29]	33.6	58.9	34	23.4	45.6	57	39.8	12	39.7	51.3	21.1
MEAA [30]	31.3	53.5	38	17.8	38.5	69.9	38.2	23.8	38.3	58.1	14.6
ATF [31]	41.9	67	27.4	36.4	41	48.5	42	13.1	39.2	75.1	33.4
I3Net [32]	30	67	32.5	21.8	29.2	62.5	41.3	11.6	37.1	39.4	27.4
PF-ATF[33]	35.6	59.9	31.6	32.7	44.1	49.4	36.8	18.4	40.3	79.3	37.5
UMT [34]	39.6	59.1	32.4	35	45.1	61.9	48.4	7.5	46	67.6	21.4
TIA [35]	**42.2**	66	**36.9**	37.3	43.7	71.8	**49.7**	**18.2**	44.9	58.9	18.2
SSDA-YOLO [10]	30.2	76.6	33.2	**48.4**	**60.7**	41.8	39.7	8.9	**64.8**	51	**38.7**
YOLO-G	31.8	**78.9**	32.9	43.2	59.1	**86.1**	49.3	7.1	62.0	**70.3**	**38.7**
↺											
SWDA [18]	dog	hrs	bike	prsn	plnt	sheep	sofa	train	tv	mAP	
SCL [27]	12.5	33.8	65.5	61.6	52	9.3	24.9	54.1	49.1	38.1	
DM [17]	26.3	34.4	67.3	61	47.9	21.4	26.3	50.1	47.3	41.5	
CRDA [28]	24.9	48.1	78.7	63	45	21.3	36.1	52.3	53.4	41.8	
HTCN [29]	14.1	33.3	74.3	61.3	46.3	22.3	24.3	49.1	44.3	38.3	
MEAA [30]	20.1	39.1	72.8	63	43.1	19.3	30.1	50.2	51.8	40.3	
ATF [31]	18.1	33.8	88.1	60.3	42.1	7.8	30.8	61.1	58.7	41.1	
I3Net [32]	7.9	41.2	56.2	61.4	50.6	42	25	53.1	39.1	42.1	
PF-ATF[33]	19.3	25	67.4	55.2	42.9	19.5	36.2	50.7	39.3	37.8	
UMT [34]	16.6	**48.5**	59.9	60.2	50.3	**35.6**	23.2	49.4	46.9	42.8	
TIA [35]	**29.5**	48.2	75.9	**70.5**	56.7	25.9	28.9	39.4	43.6	44.1	
SSDA-YOLO [10]	29.1	40.7	**87.8**	67.4	49.7	27.4	27.8	**57.1**	50.6	**46.3**	
YOLO-G	25.5	36.8	44.5	59.9	**57.3**	18.4	**41.4**	49.2	**58.6**	44.3	

Table 5 shows that the YOLO-G model based on feature alignment constraints performs more prominently in the cross-virtual and real object detection tasks.

**Table 5:** The cross-domain detection results from Pascal VOC to Watercolor.

Method	bike	bird	car	cat	dog	person	mAP
DAF [16]	75.2	40.6	48.0	31.5	20.6	60.0	46.0
WST-BSR [36]	75.6	45.8	49.3	34.1	30.3	64.1	49.9
MAF [37]	73.4	55.7	46.4	**36.8**	28.9	60.8	50.3
Strong-Weak [18]	82.3	55.9	46.5	32.7	**35.5**	**66.7**	**53.3**
YOLO-G(ours)	**89.8**	**58.6**	**53.2**	32.2	23.9	60.8	53.1

#### Detection from small dataset to large-scale dataset

In this scenario, the experimental setting takes Cityscape as the source domain and the validation set of the large-scale autonomous driving dataset BDD 100k as the target domain. The performance of the proposed model in cross-domain detection between small sample dataset and large-scale dataset is fully tested. Because there are few train targets in BDD100k, therefore, the experimental process removes the category of the train, and the experimental results are in Table 6.

**Table 6:** The cross-domain detection results from Cityscapes to BDD 100k.

Method	person	rider	car	truck	bus	motor	bicycle	mAP
DAF [16]	26.9	22.1	44.7	17.4	16.7	17.1	18.8	23.4
SWDA [18]	30.2	29.5	45.7	15.2	18.4	17.1	21.2	25.3
CRDA [28]	31.4	31.3	46.3	19.5	18.9	17.3	23.8	26.9
Source only	30.2	32.5	61.5	**28.8**	47.1	**35.5**	31.9	33.5
YOLO-G	**33.8**	**33.5**	**63.0**	27.9	**49.4**	35.3	**33.5**	**34.6**
Target only	62.9	55.4	75.5	50.2	66.8	51.5	63.3	53.4

A small sample is an important challenge faced by deep learning models, avoiding overfitting problems is the core content, for the cross-domain transformation from small sample data to large-scale application scenarios, YOLO-G gives a good solution, Table 6 shows that under unsupervised learning conditions, the combination of GRL and YOLO can improve the generalization ability of the model. Its mAP reaches 34.6, but compared with the 53.4 mAP obtained by BDD100k large-scale training set, the YOLO-G model still has a lot of room for improvement.

### Ablation experiments

Through the above experiments, the effectiveness of the YOLO-G model in cross-domain object detection is fully displayed. YOLO-G is based on YOLOV5, which has a series of tricks that are clearly different from two-stage detection models, such as Spatial Pyramid Pooling—Fast (SPPF), mosaic data augmentation. As we pointed out earlier, both tricks allow the baseline to gain excellent performance. At the same time, the distribution of pre-trained weights and weight coefficients will also affect the training results. In order to delve into the impact of each trick on YOLO-G, we conduct a full range of experiments to verify the authenticity of the model.

#### The impact of training tricks

In this section, we mainly discuss 2 tricks used by the YOLO model, SPPF and mosaic. We make the model without SPPF and mosaic as vanilla model. Then, we verify the impact of these 2 tricks in the City → Foggy City scenario, and the relevant experi mental results are shown in Table 7.

**Table 7:** The impact of training tricks used in YOLO.

Method	person	rider	car	truck	bus	train	motor	bike	mAP
Vanilla	36.6	38.5	44	20.7	30.9	13.5	22.1	29.4	29.5
+SPPF	37.7	40.7	47.1	22.9	31.6	12.1	24.5	31.6	31
+mosaic	39.8	44.2	50.3	20.9	32	4.95	23.3	35	31.3
+SPPF	39	41.4	51.8	27.5	33.3	13.1	31.7	35.8	34.2
+mosaic									

It is clear from the Table 7 that the training effect of the Vanilla model is not ideal without any tricks, and it can be said that the performance is similar to that of DAF. With the addition of the trick, it can be seen that the detection accuracy of the model gradually increases, and SPPF increases the mAP by 0.5 mAP to 31 mAP. The mosaic increased the mAP by 0.8 mAP to 31.3 mAP. Finally, with both tricks turned on, we get that under source only, the YOLOV5-L model reaches 34.2 mAP, which is far more than the Faster RCNN model without these tricks.

Through the above experiment, it is suitable to use the YOLOV5-L model with various trick aids as the baseline, which is beneficial to improve the final detection accuracy of YOLO-G in source only setting.

#### The impact of pretrained-weight

We mentioned early that with the pre-trained weights, the model has mastered some prior knowledge of the detection object. When comparing different models, the pre-trained weights loaded by different backbone models are not consistent. Therefore, it has a certain impact on the results. To this end, we carry out comparative experiments without the support of pre-trained weights in this part to test the self-learning ability of the YOLO-G model. To speed up the model training process, this set of experiments set VOC2012 as the source domain, and Clipart as the target domain, the model is carried out without loading the pre-training weights, and the rest of the parameter settings are consistent with the previous text, and the source only, YOLO-G, and target only comparison experiments are carried out respectively, and the relevant results are as shown in Table 8.

**Table 8:** The impact of pre-trained weight.

Method	aero	bicycle	bird	boat	bottle	bus	car	cat	chair	cow	table
Source only	16.2	51.4	21.7	23.8	48.3	46.2	27.7	6.13	34.1	8.19	22.7
YOLO-G	**22.3**	**69.1**	**21.9**	**31.3**	**51.4**	**34.7**	**37.1**	**8**	**41.1**	**34.5**	**24.7**
Target only	1.4	0.9	16.7	16.2	5	24.1	24.5	1.1	34.1	10	27
↺											
	dog	hrs	bike	prsn	plnt	sheep	sofa	train	tv	mAP	
Source only	6	24.7	35.5	42.3	**49**	15	**27.4**	16.3	44.8	28.4	
YOLO-G	**12.9**	**37.7**	**43.7**	**48.6**	47.8	**29.1**	25.9	**24.4**	**40.1**	**34.3**	
Target only	0.3	7.1	3	64.8	32.6	18.6	14.2	6.5	22.1	16.6	

**Fig 3:**
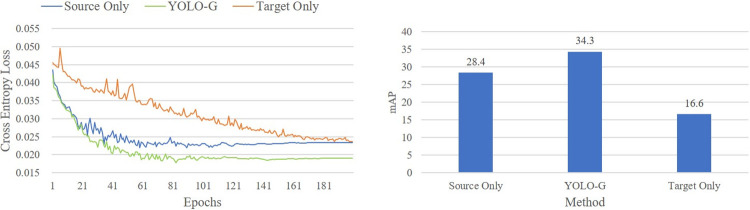
The loss curves and results of the three models trained from VOC to Clipart.

The pre-trained weights are the result of sufficient training in a very large-scale dataset, with the help of which the model can quickly extract the main features of the target in the initial stage, accelerating the speed of model convergence. Table 8 shows that the YOLO-G can quickly and accurately learn the main features of the target in cross-domain scenarios without the help of pre-trained weights. The loss curve of the training process is shown in Fig 3.

#### The impact of weight coefficients

The training loss function of the YOLO-G model consists of two parts, to test the influence of feature alignment loss on the overall detection precision of the model, under the condition of using pre-training weights, the cross-domain object detection experiment from VOC2012 to Clipart is carried out, and the α is set to 0.1, 0.5, 1.0, 1.5, and 2.0 for group experiments, and the detection results of each category are shown in Table 9.

**Table 9:** The impact of different hyper-parameter α.

weights	aero	bicycle	bird	boat	bottle	bus	car	cat	chair	cow	table
0.1	32.3	75.9	27.6	42.6	49.3	58.7	31.9	10.2	51.8	65.2	38.1
0.5	34.6	66.7	28.1	41.4	55.8	70.7	36	4.38	51.1	57	35.8
1	39.9	82	32.4	35.9	61.4	67.3	41.4	8.18	64.6	64.8	42.2
1.5	32.6	77.4	29.2	34.6	55.1	63.7	38.9	8.87	56.3	52.7	40.7
2	27.5	72.6	26.4	35.3	48	61	33.9	11.8	56.5	56.3	38
↺											
	dog	hrs	bike	prsn	plnt	sheep	sofa	train	tv	mAP	
0.1	17.2	38.5	57.5	56	56.1	34.7	33.4	37.6	46.1	43	
0.5	19	40.7	64.5	53.9	66.9	27	40.1	46.8	50.1	44.5	
1	15.7	45.1	59.6	59.1	64.8	25.1	45	45.6	59.1	**48**	
1.5	18.5	38.6	69.9	56.3	62.3	30.3	40.5	44.9	42.2	44.7	
2	16.4	36.2	78.8	58	60.3	27.1	43.3	34.4	54	43.8	

**Fig 4:**
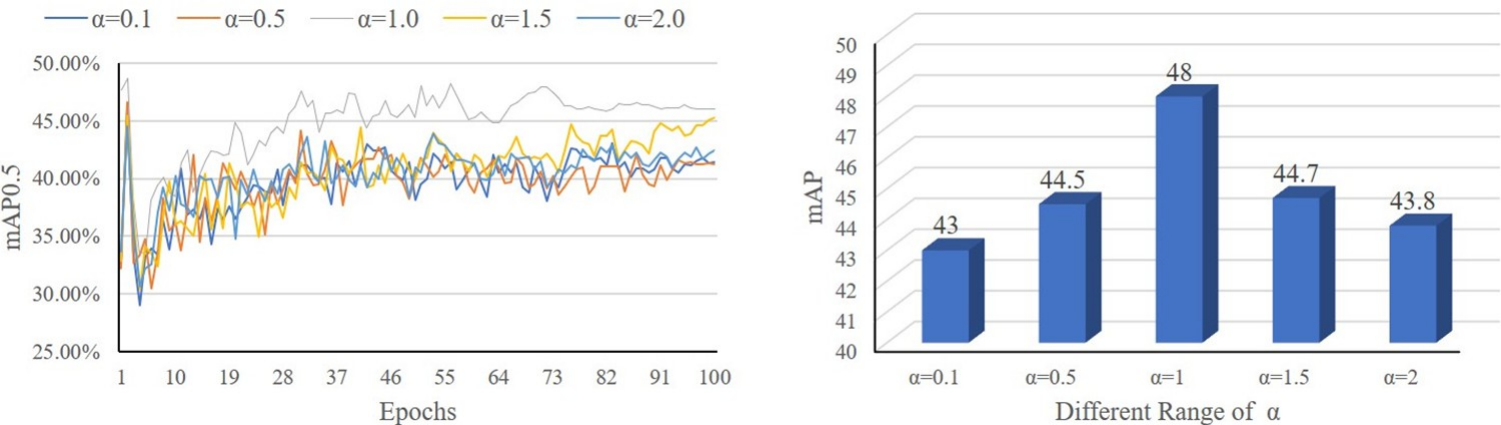
The loss curves and results of the model with 5 different α.

Comparative experiments show that the model achieves better detection precision when α is 1.0. The curve of mAP during the training process is shown in Fig 4. The left image shows that at a weight of 1, the detection accuracy can reach a higher level faster and more consistently. Therefore, the constraint weight of α of 1.0 is used for all the experiments.

## Discuss and conclusion

YOLO-G gains much stronger ability in the cross-domain object detection, summarizing all these experiments. But there is a serious problem as well, YOLO-G shows a poor performance considering small objects. YOLO is an anchor-based model, so there exists conflict when deciding which anchor is much suitable for all the objects. Especially, when there are few small objects in the dataset, the model may be dominated by the larger and easily detected objects, without the help of focal loss. In summary, there is a lot to further explore heading for the real applications.

To alleviate the problem of cross-domain object detection, this paper analyzes the characteristics of mainstream algorithm models, and proposes a simple and efficient YOLO-G model based on the YOLOV5. By introducing feature alignment branch and adversarial training, we improve the consistency of the backbone model in extracting target features, enhance the generalization of the model, and achieve better cross-domain detection ability. We also organize 9 groups of cross-domain comparative experiments, and the YOLO-G model proposed in this paper achieves precision beyond a series of SOTA models, indicating that it has better application prospects in cross-domain object detection tasks.
